# Biomarkers related to gas embolism: Gas score, pathology, and gene expression in a gas bubble disease model

**DOI:** 10.1371/journal.pone.0288659

**Published:** 2023-07-13

**Authors:** Alicia Velázquez-Wallraf, Maria José Caballero, Antonio Fernández, Mónica B. Betancor, Pedro Saavedra, Holden W. Hemingway, Yara Bernaldo de Quirós

**Affiliations:** 1 Veterinary Histology and Pathology, Atlantic Center for Cetacean Research, University Institute of Animal Health and Food Safety (IUSA), Veterinary School, University of Las Palmas de Gran Canaria (ULPGC), Canary Islands, Spain; 2 Faculty of Natural Sciences, Institute of Aquaculture, University of Stirling, Stirling, United Kingdom; 3 Department of Mathematics, University of Las Palmas de Gran Canaria (ULPGC), Canary Islands, Spain; 4 Department of Integrative Physiology, University of Colorado Boulder, Boulder, CO, United States of America; Bangladesh Agricultural University, BANGLADESH

## Abstract

Fish exposed to water supersaturated with dissolved gas experience gas embolism similar to decompression sickness (DCS), known as gas bubble disease (GBD) in fish. GBD has been postulated as an alternative to traditional mammals’ models on DCS. Gas embolism can cause mechanical and biochemical damage, generating pathophysiological responses. Increased expression of biomarkers of cell damage such as the heat shock protein (HSP) family, endothelin 1 (ET-1) or intercellular adhesion molecule 1 (ICAM-1) has been observed, being a possible target for further studies of gas embolism. The GBD model consisted of exposing fish to supersaturation in water with approximately 170% total dissolved gas (TDG) for 18 hours, producing severe gas embolism. This diagnosis was confirmed by a complete histopathological exam and the gas score method. HSP70 showed a statistically significant upregulation compared to the control in all the studied organs (*p* <0.02). Gills and heart showed upregulation of HSP90 with statistical significance (*p* = 0.015 and *p* = 0.02, respectively). In addition, HSP70 gene expression in gills was positively correlated with gas score (*p* = 0.033). These results suggest that gas embolism modify the expression of different biomarkers, with HSP70 being shown as a strong marker of this process. Furthermore, gas score is a useful tool to study the abundance of gas bubbles, although individual variability always remains present. These results support the validity of the GBD model in fish to study gas embolism in diseases such as DCS.

## Introduction

The circulation of gas bubbles through the vascular system is known as gas embolism. It can be developed under different conditions, including the accidental introduction of gas during surgical and medical procedures [1,2], penetrating traumas [3], as well as in pathological processes such as barotraumas and decompression sickness (DCS) that can produce intra- and extravascular gas bubbles [4]. Depending on the cause of gas embolism, the abundance and distribution of gas bubbles may differ [1]. DCS is mostly described in human divers [5], although it has also been reported in cetaceans [6,7] and marine turtle [8]. Fish experienced a gas embolism similar to DCS named gas bubble disease (GBD) [9,10].

GBD and DCS share similarities in terms of pathophysiology, the latter has previously been postulated as an experimental model for studying DCS [10–13]. Velázquez-Wallraf et al. [14] reported the study of GBD in fish as an alternative to traditional mammals’ models for the study of gas embolism and DCS in accordance with the replacement principle from the European regulations for the use of laboratory animals (Directive 2010/63/EU of the European Parliament).

GBD occurs in fish when the water where they inhabit gets supersaturated of dissolved gases [15]. This disease has been responsible for high fish mortalities mainly described in large hydroelectric projects where, due to the force exerted by the water mass falling from one dam to another, atmospheric gases are entrained and forced to dissolve [10]. Exposure of fish to these supersaturated waters causes the development of intravascular and extravascular gas bubbles [11], which varying in severity depending on the total dissolved gas (TDG) values and length of exposure [15]. TDG supersaturation values higher than 120% have triggered acute GBD and lead to death of some fish [15].

Gas embolism can cause mechanical and biochemical damage [16], generating pathophysiological responses, notably from endothelial cells, the first cell line to encounter intravascular gas bubbles [17]. Regarding GBD, Speare et al. (1991) [18] were the first to describe GBD lesions related to endothelial damage. Although the mechanism remains unclear, two possible pathways of endothelial cell activation have been hypothesized [18]. Direct activation by contact of the gas bubbles with the endothelial wall cells themselves has been proposed [19] and, on the other, indirect activation by the formation of molecules secondary to gas embolism [20]. Regarding direct activation, gas bubbles are foreign surfaces that cause a circulating blood-bubble interface [21]. This foreign interface may generate activation of plasma proteins, platelet aggregation, leading to thrombogenesis [22] and consequent thrombocytopenia [23], involving cell adhesion molecules, such as intercellular adhesion molecule 1 (ICAM-1). ICAM-1 is expressed by endothelial cells in response to the call of proinflammatory cytokines, facilitating the migration of leukocytes across the endothelium into inflamed tissue [24]. In addition, endothelial cells respond to bubble contact by generating biomarkers of endothelial stress, such as the heat shock protein (HSP) family [17]. The consequent endothelial dysfunction causes a decrease in nitric oxide (NO), a vasodilator molecule, that generates a relative increase in vasoconstriction factors, mainly endothelin-1 (ET-1). Therefore, activation of the expressed biomarkers due to the above circumstances represents an important target for further investigation of gas embolism [25].

In this study, we tested the hypothesis that GBD in fish induces similar vascular and cellular responses to other gas embolisms, such as DCS in mammals, and if it can be used as an alternative experimental model. To test this hypothesis, we first induced a severe GBD in a group of goldfish (*Carassius auratus*), we evaluated tissue damage through pathology, we assessed the presence, distribution, and amount of gas bubbles in several intra- and extravascular locations and analyzed the expression of different biomarkers that have previously been associated with the consequent biochemical damage of gas embolism in other laboratory animals.

## Material and methods

### Experimental fish

For this study, 20 goldfish (*Carassius auratus*), 10 males and 10 females (weight: 116.1 ± 11.3 g; length: 17.2 ± 0.5 cm long), were purchased from Tropical Centre (ICA Canarias). Fish were kept in tap water conditioned with JBL Biotopol and JBL Denitrol, according to the manufacturer’s instructions, with a natural photoperiod of 12:12h light:dark cycle and fed twice daily. Using a colorimetric test kit, water parameters such as nitrate and nitrite concentration (<10 mg/dm^3^ nitrate, 0 mg/dm^3^ nitrite), pH (±6.8), total hardness (80–300 mg/dm^3^) and chlorine (0 mg/dm^3^) were measured every two days, along with measurement of temperature (23–25°C) and dissolved oxygen (>6.0 mg/ dm^3^).

### Experimental procedures

Fish were evenly divided into two treatment groups: control group (n = 10) and GBD group (n = 10). Fish in the GBD group were individually introduced into a pressurized aquarium for 18 hours with supersaturated water produced following Velázquez-Wallraf et al. [14]. This supersaturated water was produced using a pressure vessel (max. 3 ATA) coupled to a pressurized aquarium (max. 0.5 bar) through constant recirculation of the water. Briefly, the vessel was filled with tap water at ambient pressure, the water was recirculated throughout the circuit with the help of a motor pump and pressurized synthetic atmospheric air was injected until reaching the maximum pressure of the circuit. At that time, the water was kept in constant recirculation, passing through a dissolution tube composed of small-diameter porous materials that forced the gas to dissolve in the water, until a dynamic equilibrium between the liquid phase and the gas phase was obtained. This entire process was controlled by a TDG sensor with real-time values.

Fish were introduced in the pressurized aquarium when TDG supersaturation levels of 169 ± 5% were reached in agreement with Velázquez-Wallraf et al. [14]. Fish from the control group were placed in the same aquarium but without exerting pressure or recirculation of water, so the TDG saturation level was 100% during the 18 hours of control test. During the exposure, activity and clinical signs were monitored and registered, paying special attention to opercular movements frequency, swimming behavior, presence of gas bubbles in fins or eyes, loss of scales, hemorrhages, or subcutaneous emphysema, as described by Velázquez-Wallraf et al. [14]. After 18 hours, fish from both groups were euthanized with 2-phenoxyethanol (0.6 ml/L).

### Gas score: Presence, amount, and distribution of gas bubbles

Before dissection, the eyes, integument, gills, and fins were examined under a stereo microscope for the presence of gas bubbles and lesions. Later, the coelomic cavity was carefully opened to enable the visualization of internal organs and vasculature, and presence, amount, and distribution of intravascular and extravascular gas bubbles, was evaluated using a gas score index [26] adapted to fish. The locations used for the assessment of intravascular gas bubbles were fin, opercular, cranial and caudal subcutaneous, swim bladder and posterior cardinal veins, as well as ventral and dorsal aorta. Intravascular locations were graded from 0 to 6, while extravascular locations were graded from 0 to 3 (**Table 1**), In the extravascular locations, the gas score was based on the presence of emphysema in the visceral fat and fins. Total gas score was calculated by the summation of the gas score index for each location. Gas score ranged from 0 to 54.

**Table 1:** Definition of gas score index. Gas score definition for post-mortem examinations, following Bernaldo de Quirós et al. [25].

GAS SCORE	DEFINITION
***INTRAVASCULAR LOCATIONS***	
**GRADE 0**	Absence of bubbles
**GRADE 1**	Occasional bubble
**GRADE 2**	Few bubbles
**GRADE 3**	Few bubbles and discontinuities of blood
**GRADE 4**	Moderate presence of bubbles
**GRADE 5**	Abundant presence of bubbles
**GRADE 6**	Complete sections filled with gas
***EXTRAVASCULAR LOCATIONS***	
**GRADE 0**	Absence of gas
**GRADE 1**	Scarce presence (1 organ affected)
**GRADE 2**	Moderate presence of gas (more than 1 organ)
**GRADE 3**	Abundant presence of gas (systemic)

### Gross and histopathological *evaluation*

Simultaneously to the gas score, external and internal organs were examined macroscopically for lesions, and representative samples were collected and fixed in 10% buffered formalin. Samples were processed routinely, embedded in paraffin wax and 4-μm-thick sections were cut and stained with hematoxylin and eosin (H&E) [27] and examined by two pathologists. Additionally, samples were also pretreated with chromic acid to fix lipids prior to paraffin-embedding [28]. Lipids were stained with Oil O Red and the tissue was counterstained with Mayer’s hematoxylin to discriminate between gas and fat emboli [29].

### Gene expression analysis by real-time qPCR

Molecular studies of biomarkers of vascular damage were carried out in 6 fish from each group. Samples of posterior kidney, gills, heart, and ventral aorta were collected in cryotubes with 1 ml of RNA-later (Sigma-Aldrich, Dorset, UK), maintained for 24 hours at 4°C and then preserved at -80°C until RNA extraction from the samples was performed. Sequences corresponding to the open reading frame (ORF) for selected genes, HSP70, HSP90, E-1, and ICAM-1, for *Carassius auratus* were aligned using NCBI BLAST sequence alignment analysis (NIH) and primers designed on common conserved regions (**Table 2**). In addition, elongation factor 1-alpha (EF1α) and beta-2 microglobin (B2M) were chosen as reference (housekeeping) genes, according to GeNorm.

**Table 2:** Target and reference genes. Sequences of designed primers and the efficiency and correlation values of each gene.

GEN	PRIMER	SEQUENCE	STANDARD CURVE QUALITY
**HSP70**	FORWARD PRIMER REVERSE PRIMER	ACCTACTCAGACAACCAGCC CCACTGCCGACACATTTAGG	R^2^ = 0.992 E = 106.7%
**HSP90**	FORWARD PRIMER REVERSE PRIMER	GCTTCGAGGTGCTGTACATG TTGGCCTTGTCTTCCTCCAT	R^2^ = 0.998 E = 101.2%
**ET-1**	FORWARD PRIMER REVERSE PRIMER	AGCGCTCAGTAACAGAACCT CGTTGTCTGTTTGTCTGCCA	R^2^ = 0.99 E = 101.51%
**ICAM-1**	FORWARD PRIMER REVERSE PRIMER	GGCAGTATCAGCTCCAGTGT CACACCAGTACTGAGCTCCA	R^2^ = 0.98 E = 105.74%
**EF1α**	FORWARD PRIMER REVERSE PRIMER	GATTGTTGCTGGTGGTGTTG GCAGGGTTGTAGCCGATTT	R^2^ = 0.992 E = 93.48%
**B2M**	FORWARD PRIMER REVERSE PRIMER	GCCCTGTTCTGTGTGCTGTA AAGGTGACGCTCTTGGTGAG	R^2^ = 0.999 E = 103.74%

Target tissues were dissected and homogenized in tubes with 900 μl of Qiazol Lysis Reagent using a stainless-steel bead in Tissuelyser II (Qiagen, Hilden, Germany), and following the manufacturer’s instructions for total RNA extraction. Isolated RNA was quantified via spectrophotometry (Nanovue plus spectrophotometer, Biochrom Ltd., Cambridge, UK). RNA quality was estimated by visualization on 1% agarose gel with an UV transluminator (Bio-Rad, California, USA). Complementary DNA (cDNA) was synthesized from 10 μl of extracted RNA using the High-capacity cDNA reverse transcription kit (Thermo Fisher scientific, Massachusetts, USA), and performing reverse transcription by thermocycler. To quantify this cDNA synthesis, a Qubit fluorometer (Thermo Fisher scientific, Massachusetts, USA) was used together with a Qubit ssDNA Assay kit, performing standard curves.

Confirmation of correct amplification of selected reference and target genes was carried out previously as follows: melting-curve analysis for each primer set was conducted to further confirm the specificity of PCR amplification. To calculate the PCR amplification efficiency of each primer set, serial dilution of cDNA from the samples and no-template control were used as templates for Real-Time quantitative PCR (RT-qPCR). The standard curve was generated for the calculation of amplification efficiency (E) and correlation coefficients (R2) of each primer set. These values are listed in **Table 2** and were calculated to ensure that the efficiency is above 90% for all primer pairs.

RT-qPCR was carried out using QuantStudio 12k flex RT-qPCR system (Applied Biosystems, Warrington, UK) in 96 well-plates in triplicates (technical replicates). The total volume per reaction was set at 10 μl, containing 1 μl of cDNA sample (1:10 diluted), 5 μl of TB Green™ Premix Ex Taq™ II-Tli RNaseH Plus (Takara Bio Inc., Kusatsu, Japan), 0.2 μl ROX Reference Dye II (50X; Takara Bio Inc.), 0.2 μl of forward and reverse specific primers for each gene and 3.4 μl of ddH_2_0. In addition, no-template control (C^-^NTC) and RT negative control (C^-^RT^-^) were used as negative controls for each primer set. The amplification programs were set as an initial denaturation step at 95°C for 30 sec, followed by 40 PCR cycles: 5 s at 95°C, 1 min at 62°C for annealing temperature.

The RT-qPCR data were analyzed using Design & Analysis Software v.2.4.3 (Thermo Fisher scientific, Massachusetts, USA). Cycle threshold (Ct) values were obtained using auto baseline and applied to all amplicons of the same primer set. The fold change in expression of target genes (HSP70, HSP90, ET-1, ICAM-1) was calculated using the 2^-ΔCt^ method [30]. Gene expression of each sample was normalized with the geometric mean of RNA content of reference genes (EF1α, B2M).

### Statistical analysis

The differences in biomarker gene concentration in control fish vs fish with GBD were analysed by Mann-Whitney test. Biomarker genes that were found to be statistically significant different (*p* < 0.05) or near statistically significantly different (*p* < 0.1), the correlation of these gene concentrations and total gas score was evaluated by Spearman’s correlation coefficient. The significance level used for all statistical tests performed was *p* value < 0.05. SPSS software package (version 29.0; SPSS, IBM) and R software package (version 3.3.1; R Development Core Team) for Windows were used. Statistical power was calculated by post hoc test with a significance level of 0.05, using G*power software (version 3.1.9.6; Heinrich-Heine-Universität Düsseldorf).

## Results

### Fish behavior and clinical signs under exposure to supersaturated water

During the treatment, all fish from the GBD group showed clinical signs consistent with severe GBD such as increased opercular and swimming frequency along with the presence of gas bubbles in the fins. The presence and size of these gas bubbles increased with time spent in pressurized and supersaturated water. In the last two experimental hours, fish presented with erratic movements, loss of buoyancy along with severe hemorrhages and gas bubbles in the fins. Control fish showed no behavioral or clinical signs during observation.

### Gas score: Presence, amount, and distribution of gas bubbles

**Fig 1:**
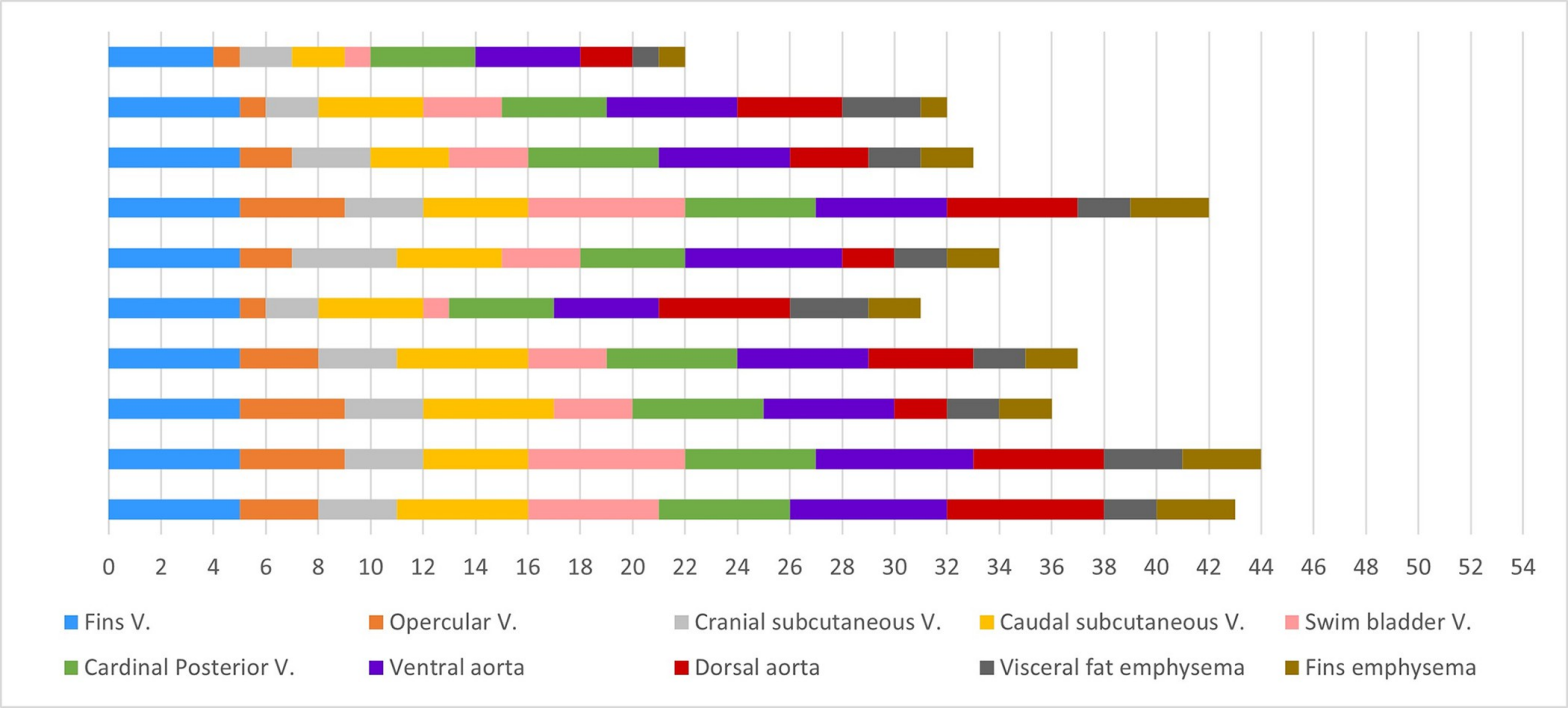
Gas score results. Gas scores obtained in the intravascular (0–6 scores) and extravascular (0–3 scores) locations of each fish in the GBD group. The total gas score of each fish is also shown.

**Fig 2:**
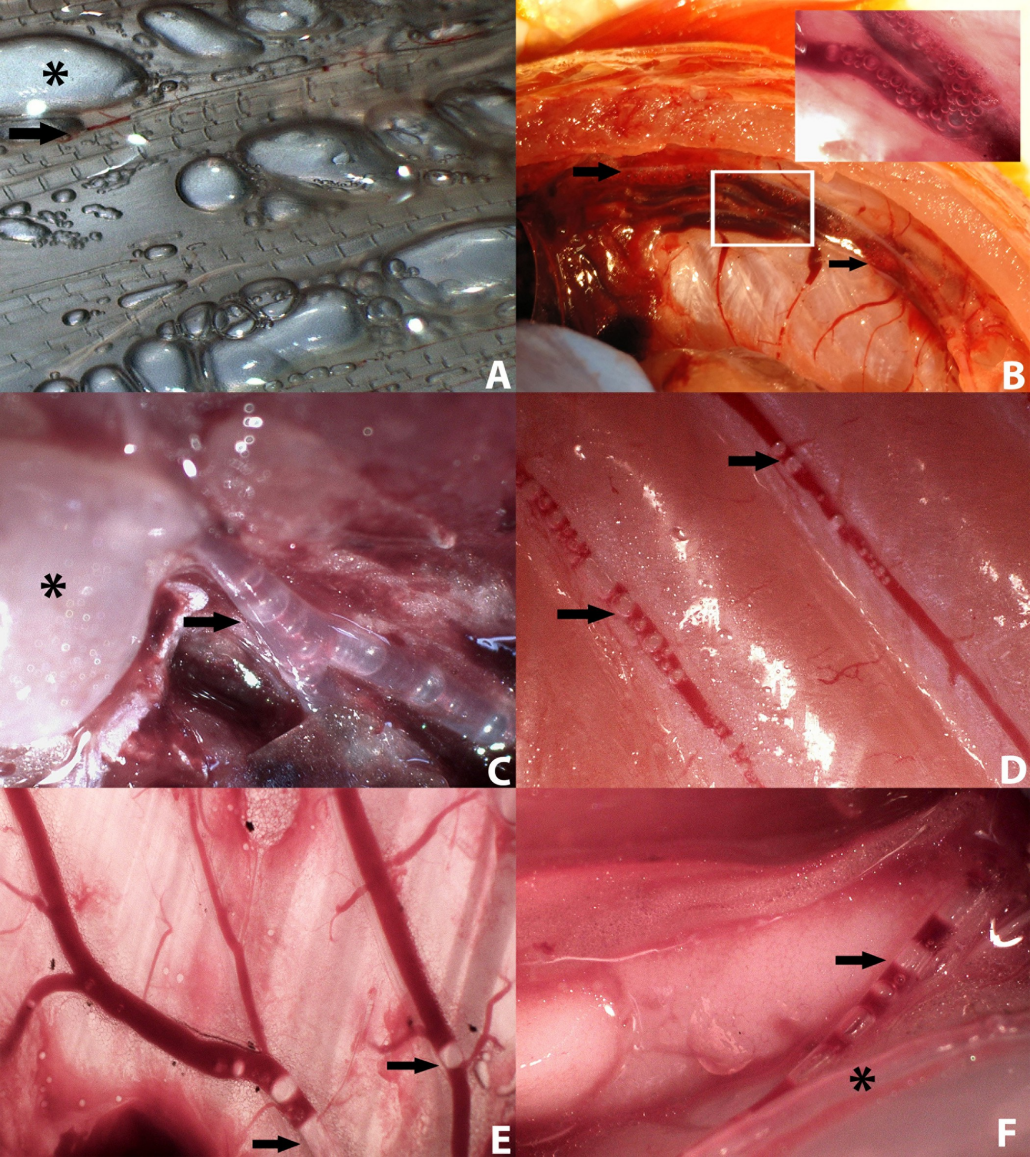
Gas score locations under stereo microscope. **A)** Fins. Presence of large gas discontinuities in the blood vessels (arrow) causing a gas score 5, together with emphysema (star), graded with gas score of 3. **B)** Posterior cardinal veins. Presence of abundant gas bubbles along their entire course (arrows), with gas score 5. Detail of gas bubbles at the beginning of the posterior cardinal veins from the caudal vein (inset). **C)** Heart and ventral aorta. Gas score 5 with abundant presence of gas bubbles at the exit of the ventral aorta (arrow) from the bulbus arteriosus (star), which appears gas dilated. **D)** Caudal subcutaneous veins with moderate presence of bubbles (arrows). Gas score 4. **E)** Cranial subcutaneous veins with few bubbles inside, although with a discontinuity (arrows). Gas score 3. **F)** The vein of the swim bladder with abundant presence of gas bubbles (arrow), running parallel to the pneumatic duct (star). Gas score 5.

The results obtained at each intravascular and extravascular location, as well as the total gas score of the GBD group are shown in **Fig 1**. In the GBD group, half of the studied intravascular locations presented a mode gas score of 5: fin veins (90% of the fish) (**Fig 2A**), posterior cardinal veins (60%) (**Fig 2B**), ventral aorta (50%) (**Fig 2C**), and dorsal aorta (30%). The subcutaneous caudal (50%) (**Fig 2D**) and opercular veins (30%) presented a mode gas score of 4; and the subcutaneous cranial (60%) (**Fig 2E**) and swim bladder (50%) vein had a mode gas score of 3 (**Fig 2F**). Within the two extravascular locations studied, mode gas score was 2, both in visceral fat emphysema (60%) and fin emphysema (50%). All the control animals showed a total gas score of 0.

### Gross and histopathological evaluation

**Fig 3:**
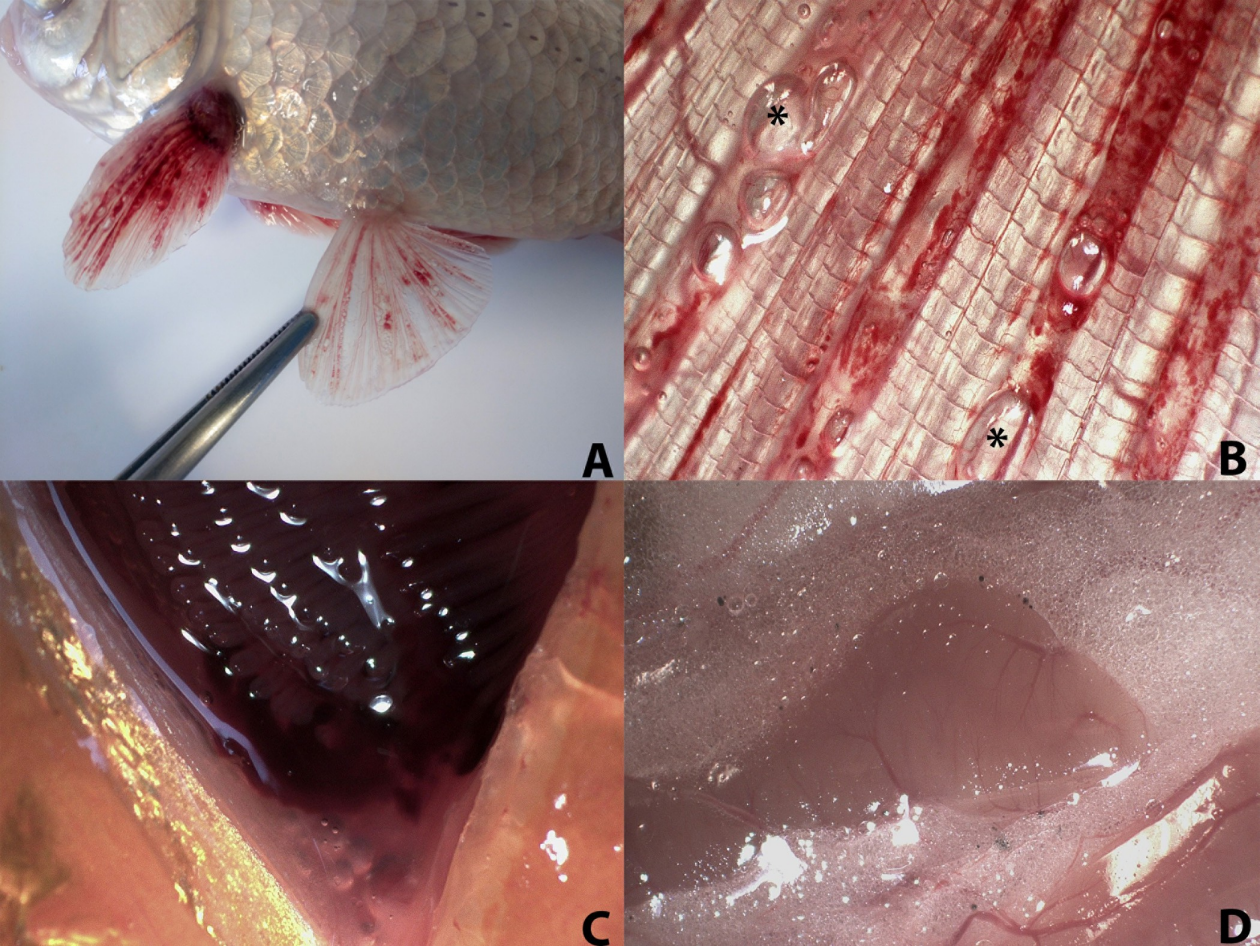
Macroscopic lesions. **A)** Presence of hemorrhages and emphysema in the fins, pectoral fins with greater affection. **B)** Fins with greater detail of the emphysema (star) and the hemorrhages described, as well as a bubble in a blood vessel. **C)** Presence of hemorrhage in the gills, appreciated in the ventral area of the gills. **D)** Portion of liver surrounded by visceral fat, which presents an emphysematous aspect.

Externally, the presence of subcutaneous emphysema was denoted by skin in 70% of fish (7/10), coinciding with areas where the loss of scales was observed in vivo. Fins presented emphysema and hemorrhages in all animals (100%, 10/10) (**Fig 3A** and **3B**), being the pectoral fins (90%, 9/10) followed by the caudal fins (50%, 5/10) the most affected locations. The gills presented severe hemorrhages (70%, 7/10) (**Fig 3C**).

In the opening of the coelomic cavity, emphysema of the visceral adipose tissue stood out in most animals (70%, 7/10) (**Fig 3D**). In addition, the swim bladder was hyperinflated (90%, 9/10) and the bulbus arteriosus of the heart appeared distended due to the presence of gas (100%, 10/10). Multiorgan congestion was observed: fins (70%, 7/10), gills (100%/10/10/10), posterior kidney (90%, 9/10), liver (90%, 9/10), spleen (70%, 7/10), and central nervous system (50%, 5/10).

**Fig 4:**
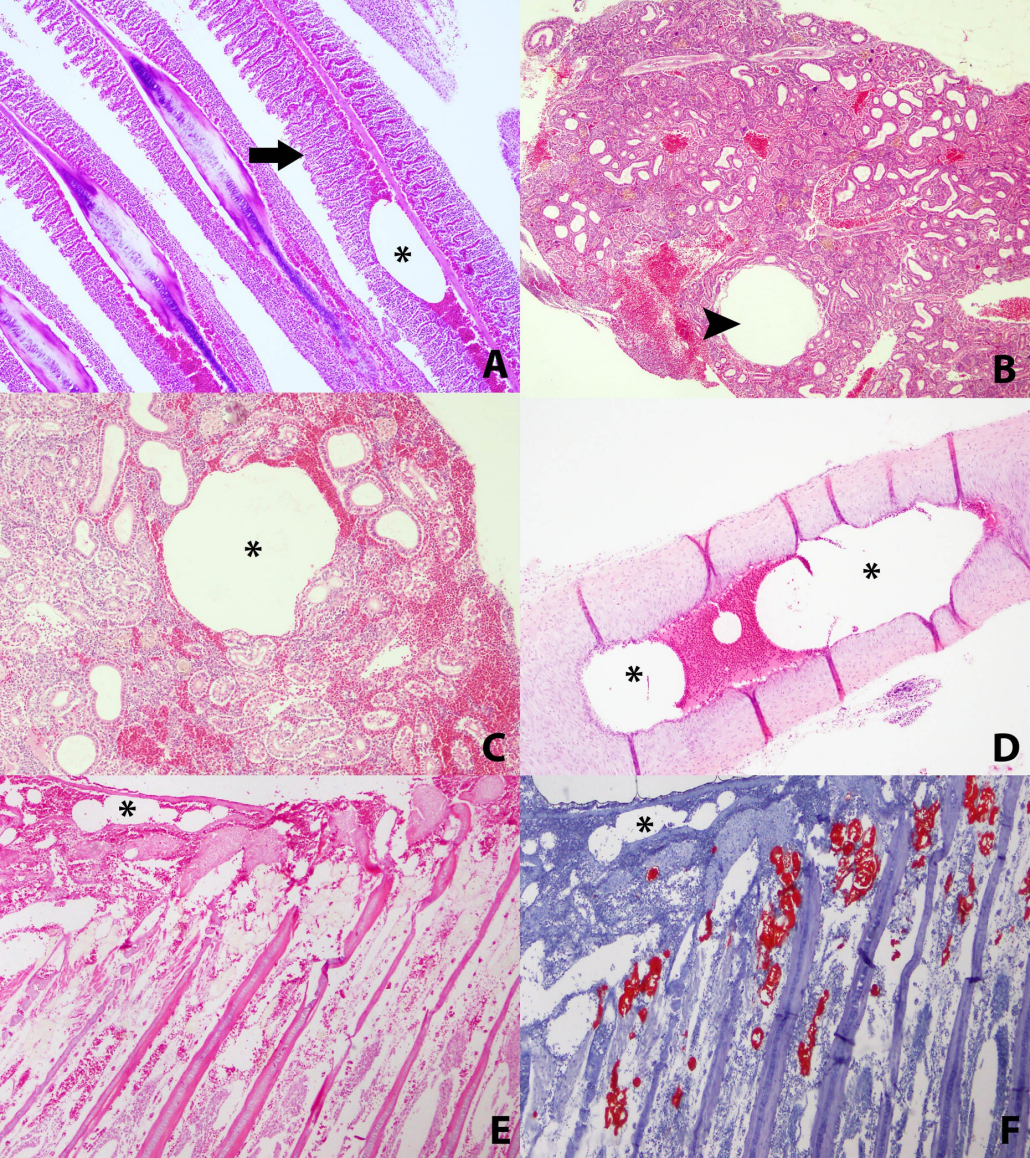
Microscopic lesions. **A)** Gills with the presence of a large gas bubble in the central vessel of the primary lamella (star). There is also fusion of the secondary lamellae (arrow). HxE x10. **B)** Posterior kidney with presence of vascular dilatations (arrowhead) and congestion of blood vessels. HxE x4**. C)** Posterior kidney with presence of large gas bubble displacing blood components to the periphery (star). HxE x10. **D)** Ventral aorta at origin of the arterial bulb, showing gas bubbles inside (star). HxE x10. **E)** Gas bubbles at the base of a holobranch (star). HxE x4. **F)** Control of gas bubbles on the same sample as above. Chromic acid x4.

Microscopically, notable findings were emphysema of fins (100%, 10/10) observed as gas-distended areas between the fin rays, together with congestion (100%, 10/10). In some animals, hemorrhages due to the rupture of larger congested blood vessels were observed in the fins (60%, 6/10). The gills showed remarkably consistent microscopic congestion (100%, 10/10) and hemorrhages (70%, 7/10), together with fusion of the secondary lamellae in some animals (50%, 5/10) (**Fig 4A**). In the posterior kidney, severely dilated blood vessels were observed (90%, 9/10), without the presence of circulating blood, while in those with the presence of blood, congestion was observed (100%, 10/10) (**Fig 4B**). Intravascular bubble-like round empty spaces among blood cells were identified in different blood vessels. These were predominantly observed in fins (90%, 9/10), gills (90%, 9/10) and posterior kidney (80%, 8/10) in considerable amounts. In other organs intravascular gas bubbles were also observed but only in some animals, such as ventral aorta (30%, 3/10) (**Fig 4D**), in the coronary veins of the heart (30%, 3/10), spleen (20%, 2/10), gonads (20%, 2/10), and liver (10%, 1/10). These structures were neither stained by H&E or Oil Red, confirming that they were gas bubbles (**Fig 4E** and **4F**). Multiorgan congestion was confirmed via microscopic visualization.

The macroscopic and microscopic pathological findings of each fish, as well as the degrees of severity shown, are described in **Tables 3** and **4**. Examination of the control group revealed no pathological findings.

**Table 3:** Macroscopic findings and degrees of severity. Macroscopic lesions were categorized as presence (X) or absence (-) and, in those cases where a severity could be attributed to the lesion, the degree of the lesion was specified, i.e., mild (Mi), moderate (Mo) or severe (S). In the total section, the presence (Yes) and the degrees of severity for each lesion were highlighted in bold.

MACROSCOPIC LESIONS									
FISH	INTEGUMENT		FINS				EYES	GILLS	
	Increased mucus production	Subcutaneous emphysema	Emphysema	Congestion	Hemorrhages	Most affected fins	Exophthalmia	Congestion	Hemorrhages
**GBD 1**	X	Mo	Mo	Mo	Mo	Pectoral, anal, and ventral	X	Mo	Mo
**GBD 2**	X	Mo	S	S	Mo	Pectoral	-	Mo	Mo
**GBD 3**	X	-	Mo	S	S	Pectoral	-	S	S
**GBD 4**	-	Mo	Mo	S	S	Pectoral and caudal	X	Mi	-
**GBD 5**	X	Mi	Mi	-	Mi	Pectoral, ventral, and anal	X	S	S
**GBD 6**	-	-	Mo	Mo	Mo	Pectoral and caudal	X	Mo	-
**GBD 7**	-	-	S	-	S	Pectoral and caudal	-	Mi	Mi
**GBD 8**	X	Mi	Mo	-	Mo	Pectoral and dorsal	X	Mo	Mo
**GBD 9**	X	Mo	Mo	Mo	Mo	Lateral, caudal, and ventral	-	Mo	Mo
**GBD 10**	X	Mi	Mi	Mi	Mi	Pectoral and caudal	X	Mo	-
**TOTAL**	Yes = 7/10 **(70%)** No = 3/10	S = 0/10 **Mo = 4/10** Mi = 3/10 Yes = 7/10 **(70%)** No = 3/10	S = 2/10 **Mo = 6/10** Mi = 2/10 Yes = 10/10 **(100%)** No = 0/10	**S = 3/10** **Mo = 3/10** Mi = 1/10 Yes = 7/10 **(70%)** No = 3/10	S = 3/10 **Mo = 5/10** Mi = 2/10 Yes = 10/10 **(100%)** No = 0/10	**P: 9/10** **(90%)** **C: 5/10** **(50%)** V: 3/10 (30%) A:2/10 (20%) L: 1/10 (10%) D:1/10 (10%)	Yes = 6/10 **(40%)** No = 4/10	S = 2/10 **Mo = 6/10** Mi = 2/10 Yes = 10/10 **(100%)** No = 0/10	S = 2/10 **Mo = 4/10** Mi = 1/10 Yes = 7/10 **(70%)** No = 3/10
**FISH**	**ADIPOSE TISSUE**	**SWIM BLADDER**	**LIVER**	**SPLEEN**	**DIGESTIVE TRACT**	**POSTERIOR KIDNEY**		**CENTRAL NERVOUS SYSTEM**	**HEART**
	**Emphysema**	**Hyperinflation**	**Congestion**	**Congestion**	**Gas-distended**	**Emphysema**	**Congestion**	**Congestion**	**Gas-distended bulbus**
**GBD 1**	Mi	X	Mi	Mo	-	Mo	S	Mi	S
**GBD 2**	Mi	X	Mi	-	-	-	Mi	Mi	S
**GBD 3**	-	X	Mo	Mo	S	S	S	-	S
**GBD 4**	Mo	-	S	-	Mo	-	-	-	Mo
**GBD 5**	Mo	X	Mo	-	-	Mi	Mi	-	Mi
**GBD 6**	-	X	Mi	Mo	-	-	S	Mo	Mo
**GBD 7**	Mo	X	Mo	Mo	Mi	-	Mo	-	S
**GBD 8**	Mo	X	-	Mo	Mi	-	S	-	Mo
**GBD 9**	Mi	X	Mo	Mi	-	Mi	Mo	Mi	S
**GBD 10**	-	X	Mo	Mi	-	-	Mo	Mi	Mi
**TOTAL**	S = 0/10 **Mo = 4/10** Mi = 3/10 Yes = 7/10 **(70%)** No = 3/10	Yes = 9/10 **(90%)** No = 1/10	S = 1/10 **Mo = 5/10** Mi = 3/10 Yes = 9/10 **(90%)** No = 1/10	S = 0/10 **Mo = 5/10** Mi = 2/10 Yes = 7/10 **(70%)** No = 3/10	S = 1/10 Mo = 1/10 **Mi = 2/10** Yes = 4/10 **(40%)** No = 6/10	S = 1/10 Mo = 1/10 **Mi = 2/10** Yes = 4/10 **(40%)** No = 6/10	**S = 4/10** Mo = 3/10 Mi = 2/10 Yes = 9/10 **(90%)** No = 1/10	S = 0/10 Mo = 1/10 **Mi = 4/10** Yes = 5/10 **(50%)** No = 5/10	S = 5/10 Mo = 3/10 Mi = 2/10 Yes = 10/10 **(100%)** No = 0/10

**Table 4:** Microscopic findings and degrees of severity. Microscopic lesions were categorized as presence (X) or absence (-) and, in those cases where a severity could be attributed to the lesion, the degree of the lesion was specified, i.e., mild (Mi), moderate (Mo) or severe (S). In the total section, the presence (Yes) and the degrees of severity for each lesion were highlighted in bold.

MICROSCOPIC LESIONS												
FISH	FINS				EYES	GILLS				LIVER	SPLEEN	
	Emphysema	Congestion	Hemorrhages	Gas bubbles	Congestion	Congestion	Gas bubbles	Hyperplasia	Hemorrhages	Congestion	Congestion	Gas bubbles
**GBD 1**	Mi	Mi	-	-	-	S	S	Mo	S	-	-	-
**GBD 2**	Mo	Mi	-	Mo	-	Mo	Mo	-	Mo	Mi	Mi	-
**GBD 3**	S	S	Mo	S	Mi	Mo	S	Mo	Mo	S	Mo	Mo
**GBD 4**	S	S	S	S	Mi	S	Mo	Mo	-	S	Mo	-
**GBD 5**	Mo	S	Mo	Mo	Mi	Mo	Mo	-	Mo	Mo	-	Mi
**GBD 6**	S	Mi	Mi	Mi	-	Mi	-	-	-	Mi	-	-
**GBD 7**	Mo	Mo	-	Mo	Mo	S	S	S	-	Mo	Mi	-
**GBD 8**	Mo	Mo	Mo	Mo	Mo	S	Mo	Mo	Mi	Mi	Mo	-
**GBD 9**	S	S	Mo	S	Mo	S	Mi	-	Mo	Mo	Mi	-
**GBD 10**	Mi	Mi	-	Mi	Mi	Mi	Mi	-	Mi	Mo	Mi	-
**TOTAL**	**S = 4/10** **Mo = 4/10** Mi = 2/10 Yes = 10/10 **(100%)** No = 0/10	**S = 4/10** Mo = 2/10 **Mi = 4/10** Yes = 10/10 **(100%)** No = 0/10	S = 1/10 **Mo = 4/10** Mi = 1/10 Yes = 6/10 **(60%)** No = 4/10	S = 3/10 **Mo = 4/10** Mi = 2/10 Yes = 9/10 **(90%)** No = 1/10	S = 0/10 Mo = 3/10 **Mi = 4/10** Yes = 7/10 **(70%)** No = 3/10	**S = 5/10** Mo = 3/10 Mi = 2/10 Yes = 10/10 **(100%)** No = 0/10	S = 3/10 **Mo = 4/10** Mi = 2/10 Yes = 9/10 **(90%)** No = 1/10	S = 1/10 **Mo = 4/10** Mi = 0/10 Yes = 5/10 **(50%)** No = 5/10	S = 1/10 **Mo = 4/10** Mi = 2/10 Yes = 7/10 **(70%)** No = 3/10	S = 2/10 **Mo = 4/10** Mi = 3/10 Yes = 9/10 **(90%)** No = 1/10	S = 0/10 Mo = 3/10 **Mi = 4/10** Yes = 7/10 **(70%)** No = 3/10	S = 0/10 **Mo = 1/10** **Mi = 1/10** Yes = 2/10 **(20%)** No = 8/10
**FISH**	**DIGESTIVE TRACT**	**POSTERIOR KIDNEY**				**CENTRAL NERVOUS SYSTEM**	**HEART**		**SPINAL CORD**	**GONADS**		
	**Congestion**	**Congestion**	**Gas bubbles**	**Vessel dilatations**	**Hemorrhages**	**Congestion**	**Congestion**	**Gas bubbles**	**Congestion**	**Congestion**	**Gas bubbles**	
**GBD 1**	-	Mi	Mi	-	-	Mi	-	-	-	-	-	
**GBD 2**	Mi	S	Mo	Mo	Mo	Mo	-	-	-	Mi	Mi	
**GBD 3**	Mi	Mo	Mi	Mi	S	Mi	S	Mi	-	Mi	-	
**GBD 4**	Mo	S	S	S	S	Mo	Mo	-	Mi	Mo	-	
**GBD 5**	Mo	S	Mo	Mo	Mo	Mi	Mo	Mo	Mi	Mi	-	
**GBD 6**	Mo	Mo	Mo	S	Mi	Mi	Mo	Mi	Mi	Mo	Mo	
**GBD 7**	Mi	Mi	S	S	-	-	-	-	-	-	-	
**GBD 8**	Mi	Mi	-	Mi	-	-	Mo	-	-	-	-	
**GBD 9**	Mi	Mi	-	Mo	Mo	-	Mi	-	-	Mi	-	
**GBD 10**	Mi	Mo	Mo	Mo	-	Mi	Mi	-	-	Mi	-	
**TOTAL**	S = 0/10 Mo = 3/10 **Mi = 6/10** Yes = 9/10 **(90%)** No = 1/10	S = 3/10 Mo = 3/10 **Mi = 4/10** Yes = 10/10 **(100%)** No = 0/10	S = 2/10 **Mo = 4/10** Mi = 2/10 Yes = 8/10 **(80%)** No = 2/10	S = 3/10 **Mo = 4/10** Mi = 2/10 Yes = 9/10 **(90%)** No = 1/10	S = 2/10 **Mo = 3/10** Mi = 1/10 Yes = 6/10 **(60%)** No = 4/10	S = 0/10 Mo = 2/10 **Mi = 5/10** Yes = 7/10 **(70%)** No = 3/10	S = 1/10 **Mo = 4/10** Mi = 3/10 Yes = 8/10 **(80%)** No = 2/10	S = 0/10 Mo = 1/10 **Mi = 2/10** Yes = 3/10 **(30%)** No = 7/10	S = 0/10 Mo = 0/10 **Mi = 3/10** Yes = 3/10 **(30%)** No = 7/10	S = 0/10 Mo = 2/10 **Mi = 5/10** Yes = 7/10 **(70%)** No = 3/10	S = 0/10 **Mo = 1/10** **Mi = 1/10** Yes = 2/10 **(20%)** No = 8/10	

### Gene expression analysis

**Fig 5:**
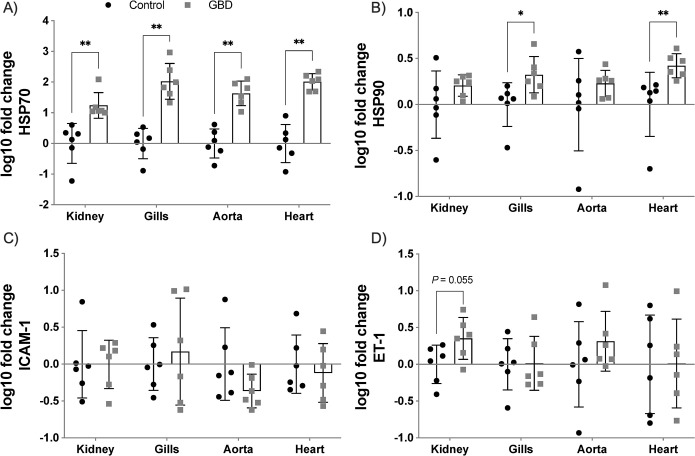
Gene expression of biomarkers in selected tissues. Log-transformed gene expression of the four biomarkers in the tissues studied from the control group and the GBD group. Statistically significant differences were observed in HSP70 for the four tissues and in HSP90 for gills and heart. Other results such as HSP90 in posterior kidney, ET-1 in posterior kidney and ventral aorta, as well as ICAM-1 in gills showed tendency to upregulation compared to the control group without reaching statistical significance.

Gene expression results were logarithmically transformed and were expressed as mean ± standard error of the mean (SEM) (**Fig 5**). The expression of HSP70 and HSP90 genes were significantly increased in gills (*p* = 0.002; *p* = 0.015, respectively) and heart (*p* = 0.002 in both). HSP70 gene expression was also significantly increased in the posterior kidney and ventral aorta (*p* = 0.002 in both), compared to the control group. ET-1 in posterior kidney and ventral aorta, HSP90 in posterior kidney, and ICAM-1 in gills showed a tendency towards upregulation compared to the control group although no statistically significant differences were observed. Results of statistical power for each biomarker expression performed are described in **S1 Table**. HSP70 expression statistical analyses in the four tissues had high statistical power (>90%).

### Correlation between gas score and genes expression

**Fig 6:**
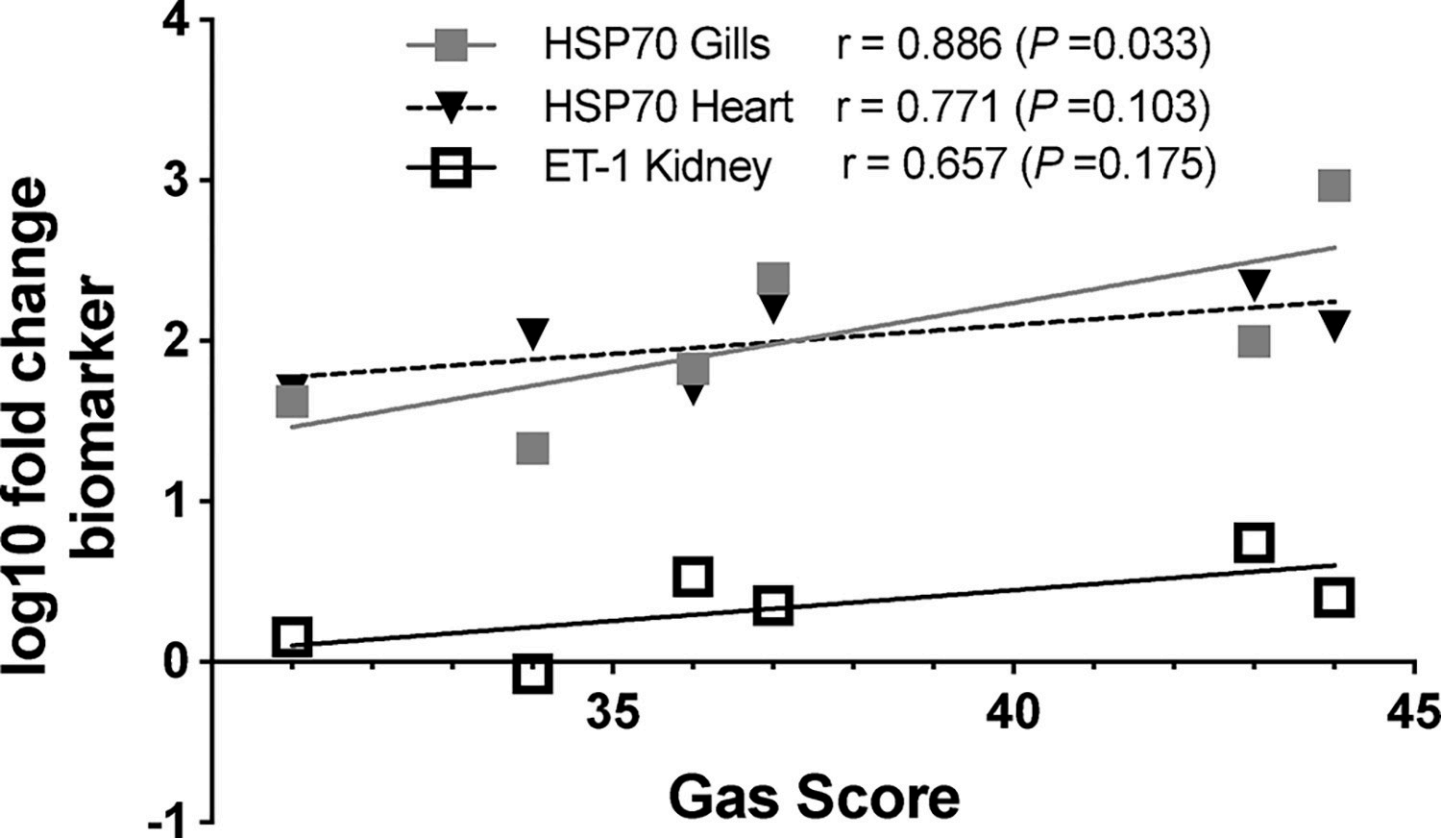
Correlation between total gas score and gene expression of biomarkers. There is a significant correlation between total gas score and HSP70 expression in gills, while ET-1 in posterior kidney and HSP70 in heart exhibit a trend but with no statistically significant correlation with total gas score.

HSP70 expression in gills correlated strongly with total gas score, with statistically significant results (*r* = 0.886, *p* = 0.033). Additionally, a tendency to correlate was found for HSP70 expression in the heart and ET-1 expression in the posterior kidney with total gas score, although these correlations were not statistically significant (*r* = 0.771, *p* = 0.103; and *r* = 0.657, *p* = 0.175 respectively) (**Fig 6**). Results of statistical power of correlation studies performed are described in **S2 Table**. HSP70 in gills and heart with total gas score had a correlation with high statistical power (>90%).

## Discussion

In the present study, we reproduced gas embolism as seen in DCS in fish (i.e., GBD) following Velázquez-Wallraf et al. [14]. The diagnosis of severe gas embolism was confirmed through a complete histopathological study and the gas score method. HSP70, HSP90, ET-1 and ICAM-1 genes were upregulated in different tissues of fish with GBD. These results were statistically significant in the case of HSP70 in the four tissues studied and in HSP90 in both gills and heart. ET-1 in posterior kidney and ventral aorta, HSP90 in the posterior kidney, and ICAM-1 in the gills had a tendency to increase their expression compared to control group, although without statistical significance. HSP70 gene expression in gills correlated positively with total gas score.

The dissolution of atmospheric gas in the water of large dams occurs continuously, due to the constant fall of water from one reservoir to another causing nearby waters to remain supersaturated [15]. Velázquez-Wallraf et al. [14], attempted to reproduce this open environment experimentally through an open aquarium, observing that gases were rapidly released to the atmosphere with a consequent loss of the TDG values of the water. They resorted to a pressurized aquarium to maintain constant recirculation of the water and stable TDG values [14]. The effect of this closed environment on the fish was measured through the control group fish, with no relevant behavioral change or clinical sign observed [14]. The control group of the present study also did not show behavioral alterations or clinical signs related to the pressurized aquarium.

The main macroscopic and microscopic findings were gas bubbles systemically distributed. Other main pathological findings included: emphysema in fins, subcutaneous tissue, and adipose tissue, hemorrhages in fins, gills, and posterior kidney, and multiorgan congestion. These pathological findings were consistent with those described previously by our group [14] and other studies of GBD in fish [31–35].

All fish in the GBD treatment group had a relatively high total gas score, although there was some inter-individual variability. This inter-individual variability was observed in the opercular veins, swim bladder vein, caudal subcutaneous veins, or the dorsal aorta. Hence, these locations were considered the most relevant to evaluate the severity of the gas embolism. Individual variability has been postulated to play a determining role in diseases that produce gas embolism [36,37].

Increased amount of gas bubbles in vascular locations systemically distributed have been also reported in DCS experimental models: in guinea pigs [38], dogs [39,40], mice [41], rabbits [26,36,42,43], sheep [44], swine [45–47], rats [17,37,48–50], and in natural occurring DCS in humans [51,52], cetaceans [7,53–56] and sea turtles [8]. The gas score index was validated as a method to evaluate the presence of gas bubbles postmortem by correlating this index with the amount of gas bubbles seen by ultrasound in vivo in the right heart of rabbits [57]. The gas score has been used as a diagnostic tool for gas embolism [26,55,58], with the affected animals showing high total gas scores, in agreement with the present study.

Circulating intravascular gas bubbles trigger activation of the vascular endothelium, widely described in diseases such as DCS [59,60] and GBD. For example, Speare et al. [18] related for the first time that GBD lesions were associated with endothelial damage. This generates a stress response of the organism, causing the emergence of different biomarkers [61].

HSPs are a superfamily of proteins that regulate different physiological processes mainly related to other proteins [62]. As a defense mechanism, the expression of these proteins can be increased under different stresses, especially thermal, oxidative, or hypoxic [63]. The main role of HSP70, one of the most studied HSPSs, is cytoprotection [64] and participation in the cell growth by mediating the production of nascent proteins [65].

HSP70 expression in gills, heart, posterior kidney, and ventral aorta showed a statistically significant pattern of upregulation in the GBD group. The gills were the location with the greatest magnitude difference between groups. The gills are the tissue that functionally resembles the lung in mammals [66]. Considering this, our results are in agreement with previous studies that showed an increase in HSP70 expression in lung, liver, and heart of rabbits [19], and in lungs of rats [61]. HSP70 expression in tissues has been postulated by several studies as a stress biomarker [67–69].

HSP90 showed a statistically significant upregulation in gills and heart. HSP90 is mainly a constitutive protein that, in certain circumstances, is induced to regulate client proteins in response to damage [70]. HSP90 expression following decompressive stress was not significantly elevated in tissues in contrast to the expression of HSP70 in the few existing studies measuring this marker, probably because baseline tissue expression levels of HSP90 in physiological situations are already high [25,71]. In this study, the significant upregulation of HSP90 in gills and heart might be explained by the severity of the gas embolism.

Zhang et al., [72] suggested that elevated levels of ET-1 in blood serum after decompressive stress might be used to evidence endothelial stress in DCS. ET-1 is the most potent vasoconstrictor factor known and it is secreted mainly by endothelial cells [73]. Although vascular endothelium is the most abundant source of ET-1 in the organism [74], this peptide is also secreted by the tubular cells of the renal medulla [75]. The posterior kidney is a highly vascularized organ, lodging in the perirenal area large caliber blood vessels such as posterior cardinal veins or the dorsal aorta [76], but also with small caliber arterioles and venules from malpighian corpuscle, where gas bubbles can easily get trapped and cause mechanical and biochemical damage. In our study, ET-1 tended to increase its expression in the posterior kidney and ventral aorta of GBD fish, but it was not statistically significant. These results could be used as a premise to test if ET-1 is elevated in blood serum of fish with GBD, and if this marker would be the best marker of gas embolism induced endothelial damage as suggested by Zhang et al. [72], since markers in blood serum accumulate over a course time if the half-life of the molecule is relatively high. This accumulation effect might provide a larger magnitude of difference between groups compared to expression alone in tissues. Therefore, the tendency to upregulation shown in both posterior kidney and ventral aorta for this biomarker is to be expected.

In the present study, no significant differences in ICAM-1 expression were found in the GBD group, in contrast to other studies [72,77]. ICAM-1 is a transmembrane protein that is mainly located in the membrane of endothelial cells and leukocytes [78], allowing the transmigration of the latter through the endothelium to inflamed tissues [79]. Increased expression of ICAM-1 in lungs of rats with gas embolism after decompression has been observed by immunohistochemistry [77]. Some authors report that increases in expression of this molecule is slight in animals with gas embolism [80] while others consider it potentially as a valid parameter for endothelial dysfunction [72]. The differences in results from our study compared to the literature may be due to the experimental design, either because of the difference in ICAM-1 detection techniques and localization (tissue/blood serum) or that our model may not reach the time necessary to produce an inflammatory response sufficient to highly express this biomarker in tissues.

Total gas score presented a statistically significant correlation with HSP70 in gills. In the case of HSP70 in heart and ET-1 in posterior kidney, there was a tendency to correlate with the total gas score, but it was not statistically significant. These organs are highly perfused; therefore, the greater the amount of gas bubbles circulating through the vascular structures, the greater the expression of biomarkers associated with stress and endothelial dysfunction should be observed. ET-1 in blood serum correlated positively with the amount of gas bubbles observed in rats [17].

The correlations between gene upregulation and the gas score demonstrate that the damage was produced by the gas bubbles. In the case of the remaining correlations that did not exhibit a statistical significance, it is probably related to the limitations of this study: small sample size, the semiquantitative nature of the gas score index, and small variability in total gas score. Future studies should increase the sample size but also induce different degrees of severity of gas score so correlations between gas score and other markers can be better established. Furthermore, the gas score is a semiquantitative variable and the gene expression of markers a quantitative variable, consequently the correlation study performed cannot discriminate as much as if both variables were quantitative. Still, with these limitations, we were able to detect a statistically significant correlation between total gas score and HSP70 expression in gills, and a tendency to correlate with no statistical significance between total gas score and HSP90 in gills and heart, and ET-1 in posterior kidney. Power analysis for correlations with total gas score showed that HSP70 in both gills and heart had high statistical power (>90%) while the trend to correlation with ET-1 in the posterior kidney showed acceptable statistical power (>70%) and may be promising correlations with a larger sample size.

The limitation of the sample size also applies to those biomarkers that, showing a tendency to upregulation, did not present statistically significant differences, being starting points for future studies with a larger sample size. The power analyses indicated that significance may have been reached if sample size were larger. On the other hand, the effect size of the non-significant samples was very small, requiring an excessively large sample size to achieve statistical significance, suggesting that there were no differences for GBD vs control for those markers. Overall, our study suggests that fish with GBD might be a valid model to experimentally study gas embolism and DCS and its effects similar to other traditional laboratory animals (i.e., mice, rats, and rabbits) but in a species with lower capacity to feel pain following the 3Rs replacement principle (Directive 2010/63/EU of the European Parliament).

In conclusion, fish with severe gas embolism showed an increase in HSPs, mainly HSP70, and a positive correlation with the gas score. Our results confirmed that HSP70 is a strong marker of gas embolism as has previously been demonstrated in other animal models, validating this model for the study of gas embolism and its effects. These results validated fish with GBD as a model for further investigation of the pathophysiological pathways of gas embolism, with the possibility of extrapolating the results to other susceptible species such as cetaceans, sea turtles or humans.

## Supporting information

S1 Table Statistical power calculation of the biomarker’s expression study. (DOCX)

S2 Table Statistical power calculation of the correlation between total gas score and biomarkers expression. (DOCX)
