# Supplementary material for: Biomarkers related to gas embolism: Gas score, pathology, and gene expression in a gas bubble disease model
Source: PLoS One. 2023 Jul 13;18(7):e0288659. doi: 10.1371/journal.pone.0288659 (PMC10343061; doi:10.1371/journal.pone.0288659)
Supplement: S1 Table — (DOCX) [file pone.0288659.s001.docx]

**SUPPORTING INFORMATION**

| BIOMARKERS EXPRESSION STUDIES | | P. KIDNEY | GILLS | V. AORTA | HEART |
| --- | --- | --- | --- | --- | --- |
| HSP70 | Effect size | 2.266 | 3.744 | 3.719 | 4.224 |
|  | Power (1-β error probability) | 0.928 | 0.999 | 0.999 | 0.999 |
| HSP90 | Effect size | 0.764 | 1.483 | 0.627 | 1.593 |
|  | Power (1-β error probability) | 0.214 | 0.614 | 0.16 | 0.676 |
| ICAM-1 | Effect size | 0.006 | 0.294 | 0.954 | 0.304 |
|  | Power (1-β error probability) | 0.05 | 0.073 | 0.306 | 0.075 |
| ET-1 | Effect size | 1.29 | 0.0374 | 0.625 | 0.018 |
|  | Power (1-β error probability) | 0.5 | 0.05 | 0.159 | 0.05 |

**S1 Table.** Statistical power calculation of the biomarker’s expression study.
