# Supplementary material for: Biomarkers related to gas embolism: Gas score, pathology, and gene expression in a gas bubble disease model
Source: PLoS One. 2023 Jul 13;18(7):e0288659. doi: 10.1371/journal.pone.0288659 (PMC10343061; doi:10.1371/journal.pone.0288659)
Supplement: S2 Table — (DOCX) [file pone.0288659.s002.docx]

**SUPPORTING INFORMATION**

| CORRELATION STUDIES | HSP70 Gills | HSP70 Heart | ET-1 Kidney |
| --- | --- | --- | --- |
| Effect size | 0.941 | 0.878 | 0.8106 |
| Power (1-β error probability) | 0.998 | 0.911 | 0.719 |

**S2 Table.** Statistical power calculation of the correlation between total gas score and biomarkers expression.
